# The Oncogenic Lipid Sphingosine-1-Phosphate Impedes the Phagocytosis of Tumor Cells by M1 Macrophages in Diffuse Large B Cell Lymphoma

**DOI:** 10.3390/cancers16030574

**Published:** 2024-01-29

**Authors:** Tracey A. Perry, Navta Masand, Katerina Vrzalikova, Matthew Pugh, Wenbin Wei, Robert Hollows, Katerina Bouchalova, Mahdi Nohtani, Eanna Fennell, Jan Bouchal, Pamela Kearns, Paul G. Murray

**Affiliations:** 1 Institute of Cancer and Genomic Sciences, University of Birmingham, Birmingham B15 2TT, UK; navtamasand@gmail.com (N.M.); wenbin.wei2@durham.ac.uk (W.W.); robert.hollows@gmail.com (R.H.); p.r.kearns@bham.ac.uk (P.K.); 2 Institute of Immunology & Immunotherapy, University of Birmingham, Birmingham B15 2TT, UK; k.vrzalikova@bham.ac.uk (K.V.); m.pugh.1@bham.ac.uk (M.P.); 3 Royal College of Surgeons in Ireland Medical University of Bahrain, Manama P.O. Box 15503, Bahrain; 4 The Palatine Centre, Durham University, Durham DH1 3LE, UK; 5 Department of Pediatrics, Faculty of Medicine and Dentistry, Palacky University and University Hospital Olomouc, 77900 Olomouc, Czech Republic; katerina.bouchalova@upol.cz; 6 Limerick Digital Cancer Research Centre, Health Research Institute and Bernal Institute and School of Medicine, University of Limerick, Limerick V94 T9PX, Ireland; mahdi.nohtani@ul.ie (M.N.); eanna.fennell@ul.ie (E.F.); 7 Department of Clinical and Molecular Pathology, Institute of Molecular and Translational Medicine, Faculty of Medicine and Dentistry, Palacky University and University Hospital Olomouc, 77900 Olomouc, Czech Republic; jan.bouchal@upol.cz; 8 National Institute for Health Research (NIHR), Birmingham Biomedical Research Centre, University of Birmingham, Birmingham B15 2TT, UK

**Keywords:** S1P, SPHK1, S1PR1, DLBCL, macrophages, phagocytosis, rituximab, ofatumumab, CD20 monoclonal antibodies

## Abstract

**Simple Summary:**

Diffuse large B cell lymphoma (DLBCL), the most common form of non-Hodgkin lymphoma, is clinically aggressive and associated with poor patient outcomes. Antagonists of the small oncogenic lipid sphingosine-1-phosphate (S1P) are already in the clinic and have been suggested to have therapeutic potential in DLBCL. We have studied the impact of S1P signaling on the recruitment of macrophages and their phagocytic functions following the treatment of DLBCL cells with CD20-targeting antibodies. We have shown that tumor-derived S1P is a major chemoattractant for monocytes and macrophages, both in vitro and in animal models of DLBCL, an effect mediated by the S1P receptor S1PR1. However, S1P also robustly inhibited the phagocytosis of antibody-treated tumor cells by M1 macrophages. Future experiments could be directed toward investigating the therapeutic effects of blocking S1P–S1PR1 signaling in combination with chemotherapy and CD20-targeting antibodies.

**Abstract:**

Background: A total of 30–40% of diffuse large B cell lymphoma (DLBCL) patients will either not respond to the standard therapy or their disease will recur. The first-line treatment for DLBCL is rituximab and combination chemotherapy. This treatment involves the chemotherapy-induced recruitment of tumor-associated macrophages that recognize and kill rituximab-opsonized DLBCL cells. However, we lack insights into the factors responsible for the recruitment and functionality of macrophages in DLBCL tumors. Methods: We have studied the effects of the immunomodulatory lipid sphingosine-1-phosphate (S1P) on macrophage activity in DLBCL, both in vitro and in animal models. Results: We show that tumor-derived S1P mediates the chemoattraction of both monocytes and macrophages in vitro and in animal models, an effect that is dependent upon the S1P receptor S1PR1. However, S1P inhibited M1 macrophage-mediated phagocytosis of DLBCL tumor cells opsonized with the CD20 monoclonal antibodies rituximab and ofatumumab, an effect that could be reversed by an S1PR1 inhibitor. Conclusions: Our data show that S1P signaling can modulate macrophage recruitment and tumor cell killing by anti-CD20 monoclonal antibodies in DLBCL. The administration of S1PR1 inhibitors could enhance the phagocytosis of tumor cells and improve outcomes for patients.

## 1. Introduction

Many patients with diffuse large B cell lymphoma (DLBCL) experience long-term disease-free survival following standard-of-care treatment, which includes the anti-CD20 monoclonal antibody rituximab and combination chemotherapy (R-CHOP) [1,2]. However, around 30–40% of patients will either relapse or have disease refractory to first-line treatment [3,4,5,6]. Currently, we lack an understanding of the mechanisms that underlie this variable response to therapy.

The transformed B cells of DLBCL are surrounded by a complex milieu of different cell types including T cells, macrophages, and other stromal cells that contribute to the survival, proliferation, and immune escape of the tumor cells [7,8,9,10]. The importance of the DLBCL microenvironment is underscored by the existence of two clinically relevant gene signatures that are based on differences in stromal cell content and referred to as the ‘stromal-1’ and ‘stromal-2’ signatures [11]. The ‘stromal-1’ signature is characterized by extracellular matrix deposition and macrophage infiltration, whereas the ‘stromal-2’ signature reflects angiogenic processes and a high density of blood vessels [11]. 

Consistent with the favorable prognosis associated with the macrophage-rich ‘stromal-1’ signature, patients with high CD68-positive macrophage counts have been shown to have improved five-year progression-free and overall survival following R-CHOP, compared with patients with low macrophage counts [12]. This association appears to hold true across different patient cohorts but is not observed in patients receiving CHOP alone in the pre-rituximab era [12]. In fact, some studies show that high macrophage counts correlate with poor outcomes in patients treated with CHOP alone [12,13,14]. Thus, the prognostic influence of tumor-associated macrophages in DLBCL appears to depend on whether anti-CD20-directed antibody therapy is employed or not [15]. It is noteworthy that similar effects of macrophages in the presence of rituximab have been observed in patients with follicular lymphoma [16,17].

These studies raise the important question of how macrophages influence rituximab-based chemo-immunotherapy. Antibody-dependent cellular cytotoxicity/phagocytosis is reported to be the most important mechanism of killing of lymphoma cells by rituximab [18]. Thus, Fcγ receptors have been shown to be required for rituximab-mediated killing of lymphoma cells. Furthermore, Fcγ receptor polymorphisms that modulate the affinity of binding to IgG subclasses affect the efficacy of rituximab when used as a single agent [19,20].

Previous studies point to a pathogenic role for the bioactive lipid sphingosine-1-phosphate (S1P) in the pathogenesis of B cell lymphomas, including DLBCL. S1P is produced intracellularly by the actions of the two sphingosine kinase isoforms sphingosine kinase-1 (SPHK1) and sphingosine kinase-2 (SPHK2) [21,22]. S1P can be exported out of cells where it activates autocrine or paracrine signaling through one or more of five different S1P receptors (S1PR1–5) [23,24]. S1PRs are G-protein coupled receptors that have partially convergent or antithetic effects [25,26,27]. For example, S1PR1 couples to Gi to evoke pro-migratory effects, while S1PR2 couples to Gi, Gq, and G12/13 to inhibit cell migration [28]. Normally, tissue S1P levels are too low to activate its receptors [29]. However, during inflammation or in cancer, S1P levels rise and are sensed by infiltrating immune cells, including lymphocytes and macrophages [30]. Higher levels of S1P in tumors can be due to the elevated expression of sphingosine kinases by the cancer cells [31,32]. In particular, SPHK1 is prominently over-expressed by numerous cancer types, in many cases correlating with increased tumor grade and reduced patient survival [33,34,35,36,37]. 

Increased S1P production and aberrant S1PR expression are observed in B cell lymphoma [38,39]. For example, in the Hodgkin/Reed–Sternberg (HRS) cells of classical Hodgkin lymphoma (cHL), S1P has been shown to activate phosphatidylinositide 3-kinase (PI3-K) signaling, an effect that is mediated by the increased expression of S1PR1 and the decreased expression of S1PR2 [40]. Genes upregulated by this pathway in HRS cells included the basic leucine zipper transcription factor ATF-like 3 (BATF3), which is aberrantly expressed in cHL [40]. In DLBCL, the S1PR2 gene is either mutated or transcriptionally downregulated (for example by the EBV oncogene latent membrane protein-1), which leads to aberrant signaling including constitutive activation of PI3-K and STAT pathways [39,41,42,43]. S1P has also been shown to induce angiogenesis in DLBCL [44]. S1PR1/S1PR3-YAP and S1P-ALOX15 signaling have also recently been shown to contribute to aggressive behavior in obesity-related lymphomas [45]. 

Signaling through S1PRs also plays a crucial role in various inflammatory and immune responses. T and B lymphocytes, along with endothelial cells, exhibit distinct profiles of S1PRs, which significantly regulate their development, recirculation, tissue homing patterns, and chemotactic responses [46,47]. The impact of S1PR signaling extends to circulating monocytes, resembling the effects on lymphocytes, influencing monocyte activation, which has been shown to be mediated through CD40 expression and TNF-α production [48,49]. S1P also plays a role in lymphocyte contact and promotes the formation of endothelial junctional complexes in lymph nodes [49].

In the present study, we have explored the role of tumor-derived S1P in the recruitment of monocytes and macrophages and its effects on the phagocytosis of DLBCL tumor cells opsonized with therapeutic anti-CD20 monoclonal antibodies.

## 2. Materials and Methods

### 2.1. Human Tissues and Cell Lines

DLBCL tissues were obtained with ethical approval from Birmingham Women’s and Children’s Hospital (Birmingham, UK) and the University of Palacky, Czech Republic (REC_RG_HBRC_12-071 and REC_RG_HBRC_14-180). Leukocyte cones were obtained with ethical approval from the National Blood Service (Birmingham, UK; REC_RG_15_165). DLBCL cell lines (DSMZ, Braunschweig, Germany) were cultured according to recommended guidelines as follows: OCI-Ly1 in IMDM + 10%FBS (Gibco, Waltham, MA, USA), split 1:3 every 3 days; OCI-Ly3 in RPMI1640 + 20% FBS (all Gibco), split 1:5 every 3 days; and SUDHL6 cells in RPMI1640 + 10% FBS (Gibco), split 1:3 every 3 days. To prepare conditioned media from DLBCL cell lines, cells were diluted to 1 × 10^6^ cells/mL in fresh complete media and incubated in a humidified incubator set to 37 °C with 5% CO_2_ for 24 h. The conditioned media was collected, centrifuged at 1500 rpm for 5 min, and filtered through a 0.22 µm syringe filter. 

### 2.2. Monocyte Isolation/Macrophage Polarization 

Anti-CD14 magnetic beads (Miltenyi, Bergisch Gladbach, Germany) were used to isolate CD14+ monocytes from leukocyte cones. Monocytes were polarized to M1 (5 ng/mL hu-GMCSF) or to M2 (25 ng/mL hu-MCSF) (both Peprotech, Cranbury, NJ, USA) macrophages in RPMI-1640 + 1% GlutaMAX + 10% FBS (Gibco) for 7 days. The purity of CD14+ isolation and the phenotype of M1 and M2 macrophages were evaluated by flow cytometry. The following antibodies were used in the study: CD14 (FITC), CD68 (PE-TxRed), CD163 (APC), CD206 (PE-CY7), and S1PR1 (APC) (Thermo Fisher, Waltham, MA, USA). ELISA Duoset kits (R&D Systems, Minneapolis, MN, USA) were used to measure levels of IL-10 and IL-12(p70) released by macrophages.

### 2.3. Transwell Migration Assays

S1P (Sigma Aldrich, Burlington, MA, USA), sphingomab, and an isotype control (LPath Inc., San Diego, CA, USA) were prepared and used as before [44]. The S1PR1 functional antagonists/inhibitors siponimod (S7179) and ponesimod (S8241) (Selleckchem, Munich, Germany) were reconstituted in DMSO to a 50 mM stock concentration and diluted to working concentrations in RPMI 1640 (Gibco). For S1PR1 inhibition, cells were pre-treated with 100 nM of siponimod or ponesimod for 1 h prior to the assays. Transwell migration assays were performed in 3 µm cell culture inserts (353492 Corning, Tewksbury, MA, USA) and 24-well low-attachment plates (Corning). M1 and M2 cells (100 µL cell suspension) were added into the cell culture insert and conditioned media to the bottom of the well (600 µL). After 4 h at 37 °C, migrated cells were collected, washed, and counted.

### 2.4. Phagocytosis Assay

Dead cells were removed from DLBCL cell lines 24 h prior to assay. M1 and M2 macrophages were stained with CFSE (Becton Dickinson, Franklin Lakes, NJ, USA) and DLBCL cell lines were stained with eFLUOR-450 (Thermo Fisher) according to the manufacturer’s instructions. CFSE-positive macrophages were re-suspended in 1% FBS+RPMI-1640 (Gibco) and pre-treated with a vehicle or 1 µM S1P for 1 h at 37 °C. eFLUOR-450-positive DLBCL cell lines were re-suspended to 1% FBS+RPMI-1640 (Gibco) and pre-treated with the isotype control (human IgG, Thermo Fisher) or rituximab 1 μg/mL or ofatumumab 1 μg/mL (kind gift from Professor Tanja Stankovic, University of Birmingham) for 2 h at 37 °C. Pre-treated DLBCL cells were co-cultured at a 1:1 ratio with M1 or M2 macrophages at 37 °C for 1.5 h in the following combinations: vehicle + isotype, vehicle + rituximab or ofatumumab, S1P + isotype, and S1P + rituximab or ofatumumab. After incubation, cells were washed in cold 0.9% NaCl + 0.1% EDTA and re-suspended in cold PBS. The total number of CFSE-positive/eFLUOR-450-positive cells (representing phagocytosed cells) were analyzed by flow cytometry. A Phagocytosis Assay Kit (IgG FITC; Cayman Chemical, Ann Arbor, MI, USA) was used to measure the phagocytosis of FITC+ beads by M1 and M2 macrophages. 

### 2.5. Immunohistochemistry

DLBCL tissues were subjected to heat-mediated antigen retrieval in citric acid buffer pH6 then blocked in TNB buffer (Akoya, Marlborough, MA, USA) for 30 min. Mouse antibody F4/80 (clone CI:A3-1, MCA497GA, Bio Rad, Hercules, CA, USA) was diluted (1:100) in TNB buffer (Akoya) and incubated overnight at 4 °C. Tissues were treated with peroxidase blocking solution for 10 min and then incubated with an appropriate secondary antibody conjugated to HRP (Agilent DAKO, Santa Clara, CA, USA). Proteins were visualized using diaminobenzidine (Vector Laboratories, Peterborough, UK) and counterstained with Mayer’s haematoxylin. 

### 2.6. DLBCL Xenografts

DLBCL cell lines (OCI-Ly1 and SUDHL6) were injected subcutaneously in the flank of NOD-scid IL2Rgnull (NSG) mice (Charles River Laboratories, Wilmington, MA, USA). When tumors reached >80 mm^3^, mice were randomized into groups and treated by oral gavage every other day with siponimod (6 mg/mL, Selleckchem) or a vehicle [44], and the experiment ended once tumors reached 1000 mm^3^. A20, a mouse B cell lymphoma cell line (kind gift from Dr Martin Hogenkamp, University of Birmingham, Birmingham, UK), was cultured in RPMI1640 Hibri-Max (Merck, Burlington, MA, USA) + 10% FBS + 2 mM L-glutamine + 1 mM Hepes + 1 mM NaPyruvate + 0.5 mM 2-mercaptoethanol (all Gibco). BALB/C mice (Charles River Laboratories) were used as before [44]. Tumors were fixed in 10% neutral buffered formalin then paraffin-embedded or prepared as single cell suspensions [44]. For flow cytometry analysis, tissues were weighed and counting beads were used to determine the total number of cells per mg, which were then extrapolated to absolute numbers using the weight of the entire tissue. Human CD45-Pacific blue and mouse CD45-AlexaFluor 700, F4/80-PE, CD11b-PECy7 (Biolegend, San Diego, CA, USA) antibodies were used. Animal experiments were performed under the project license approved by the Home Office and the Animal Welfare and Ethical Review Body at the University of Birmingham (code PD303CC67).

### 2.7. Gene Expression and Statistical Analysis

We re-analyzed two publicly available datasets that reported gene expression in DLBCL. One of these datasets, reported by Reddy et al. [50], comprised 624 DLBCL samples (in.bam format) that passed quality control checks. Data were downloaded from the European Genome–Phenome Archive at The European Bioinformatics Institute (Study ID: EGAS00001002606, Dataset ID: EGAD00001003600). Aligned RNA seq data were assigned to individual genes using the featureCounts function of Rsubread. Read counts were normalized between samples and converted to counts-per-million (cpm) reads for each gene using the edgeR package: 3.14.0 [51,52]. The second dataset was reported by Morin et al. [53,54] and downloaded from the controlled access area of the NIH database of genotypes and phenotypes (dbGap; http://www.ncbi.nlm.nih.gov/gap, accessed on 7 April 2016) using accession number phs000532. Re-analysis of these data was performed as we have previously described [44,53,54]. Meta-analysis of 11 different DLBCL datasets was performed as before [55]. Tumor-associated macrophage gene expression data were kindly provided by Professor Christopher Gregory (University of Edinburgh) [56]. Gene Set Enrichment Analysis (GSEA) was performed using the Database for Annotation, Visualization, and Integrated Discovery (DAVID). In this analysis, expected versus observed genes refer to the comparison between the number of genes in a given gene set that would be statistically expected by chance alone (‘expected’) versus the actual observed number of genes from the input gene list belonging to that set (‘observed’). The analysis helps identify whether a particular gene set is significantly over-represented or under-represented in the experimental data. Data for the in vitro experiments shown were representative of at least three independent experiments. Statistical tests are indicated in relevant sections and were considered statistically significant if *p* < 0.05.

## 3. Results

### 3.1. Genes Correlated with SPHK1 Expression in DLBCL Are Enriched for Macrophage Functions

**Figure 1:**
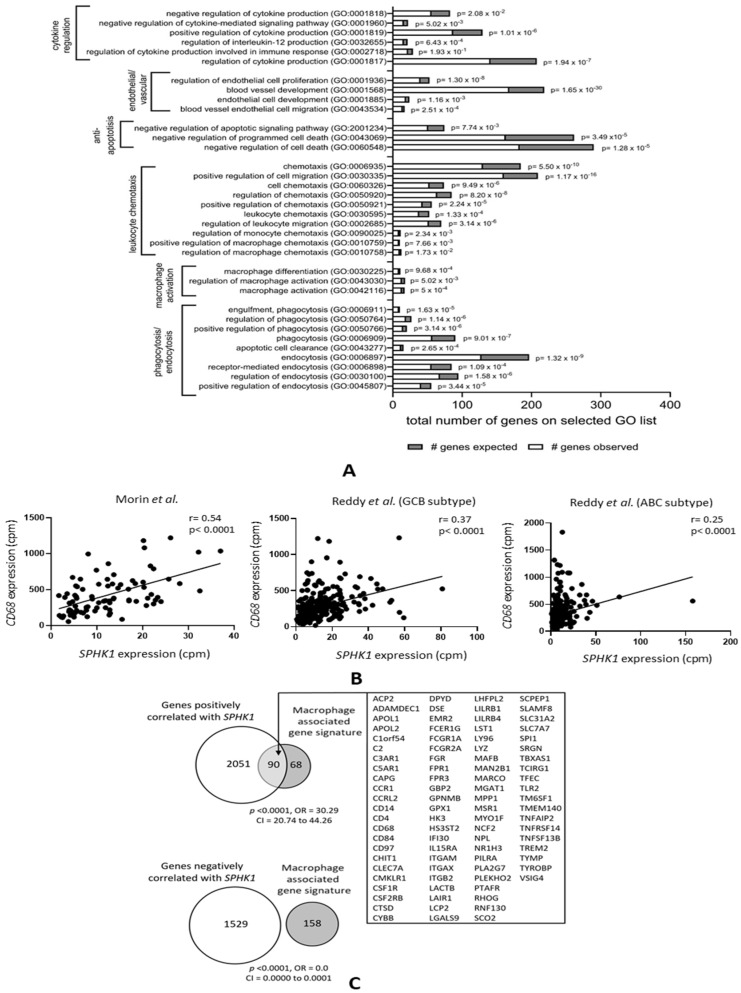
Genes correlated with *SPHK1* expression in DLBCL are enriched for macrophage functions. (**A**) GO analysis of genes the expression of which was positively correlated with *SPHK1* in >5 out of 11 datasets from DLBCL patients showing the expected enrichment of genes involved in known functions of S1P, including cytokine regulation, endothelial cell proliferation, blood vessel formation, and protection from apoptosis. Genes involved in macrophage activation, migration/chemotaxis, and phagocytosis were also significantly enriched. (**B**) Correlation between *SPHK1* and *CD68* expression in two additional datasets not included in the meta-analysis shown in (**A**). The left panel shows data from Morin et al. [53,54] and the middle and right panels show data from Reddy et al. [50] split by subtype. Correlation coefficients (r values) were calculated by the Pearson method. (**C**) Macrophage-associated signature genes were significantly enriched among genes positively correlated with *SPHK1* expression (upper panel) and depleted among genes negatively correlated with *SPHK1* expression (lower panel).

To identify the impact of S1P on the DLBCL microenvironment, we utilized our previous meta-analysis of 11 different DLBCL gene expression datasets [44,55] in which we had identified subsets of genes either positively or negatively correlated with *SPHK1* expression (that we used as a surrogate of S1P signaling). A gene ontology (GO) analysis of genes positively correlated with *SPHK1* not only revealed the expected enrichment of genes with biological activities consistent with known tumor-associated S1P functions, including anti-apoptosis, vascular and endothelial cell development, and cytokine regulation (Figure 1A), but also the significant enrichment of GO terms associated with macrophage functions, including macrophage activation, macrophage migration, and the regulation of phagocytosis. To confirm this, we used two further DLBCL datasets not used in our original meta-analysis [50,53,54]. Figure 1B shows that, in these additional datasets, *SPHK1* mRNA was positively correlated with expression of the macrophage marker gene *CD68*, a relationship also observed when the tumors were split into germinal center B cell (GCB) and activated B cell (ABC) subtypes, although this association was somewhat weaker for the ABC subgroup. We also investigated the overlap between genes positively correlated with *SPHK1* expression [44,55] and those comprising a macrophage-associated gene expression signature derived from different primary tumor types, including DLBCL [56]. We found a significant enrichment of the macrophage-associated signature genes among the set of genes positively correlated with *SPHK1* expression (odds ratio (OR) = 30.29; *p* < 0.0001) and their significant depletion among genes negatively correlated with *SPHK1* expression (OR = 0.0; *p* < 0.0001) (Figure 1C). While such an association could be explained by the expression of SPHK1 in tumor-associated macrophages, our previous immunohistochemistry of DLBCL shows that SPHK1 is predominantly expressed by tumor cells [44].

### 3.2. S1P Mediates the Migration of Human Monocytes to DLBCL Cells In Vitro

**Figure 2:**
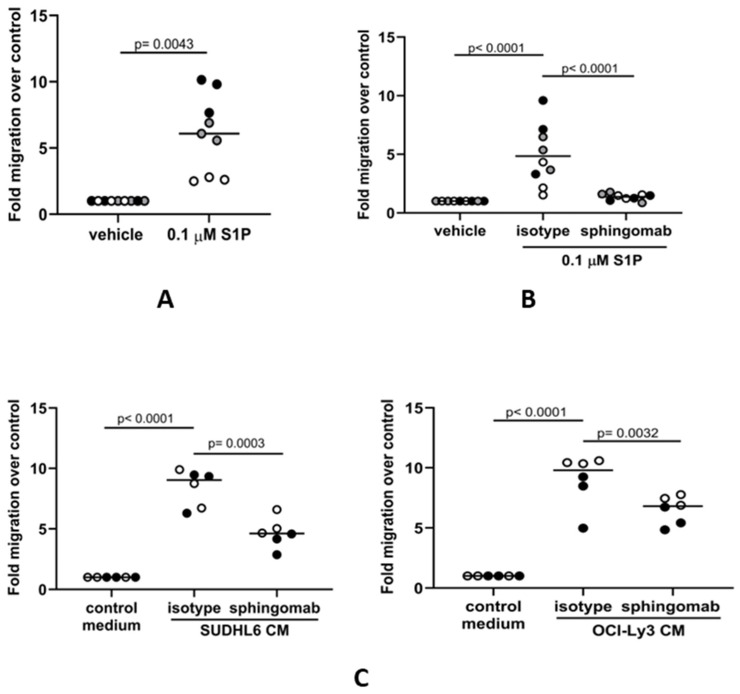
S1P mediates the migration of human monocytes to DLBCL conditioned media in vitro. (**A**) Transwell assay showing that the migration of CD14+ monocytes is significantly increased in the presence of 0.1 µM S1P. (**B**) S1P-induced migration is blocked in the presence of the S1P neutralizing antibody sphingomab (0.1 µg/mL) but not by an isotype control. (**C**) Monocyte migration to conditioned media (CM) from GCB (SUDHL6) and ABC (OCI-Ly3) DLBCL cell lines was inhibited in the presence of sphingomab. Data show three (**A**,**B**) or two (**C**) donors in triplicate and are representative of three independent experiments. The individual donors are indicated by grey, black, and white circles; data means are shown by solid bars. *p*-values were calculated using a paired *t*-test (**A**) and two-way ANOVA (**B**,**C**).

We next studied if the association between SPHK1 and the expression of genes associated with macrophage functions could be explained by the S1P-mediated migration of monocytes to the tumor. To do this, we isolated human CD14-positive monocytes from the blood of healthy donors and measured their migration to medium containing either S1P or a vehicle as a control. We found that S1P significantly increased the migration of monocytes above the control (Figure 2A), confirming previous reports that S1P is a strong chemoattractant for human monocytes [57]. As a further control for the specificity of this effect, we showed that this increased migration could be inhibited by sphingomab, an S1P-specific neutralizing antibody (Figure 2B). Because we have previously shown that DLBCL cells can secrete S1P [44], we next measured the migration of human monocytes to conditioned media from DLBCL cell lines derived from a patient with GCB DLBCL (SUDHL6) or from a patient with ABC DLBCL (OCI-Ly3). Compared with controls, the migration of monocytes was significantly increased to media conditioned by each of the cell lines (Figure 2C) and was blocked by sphingomab. We conclude that the migration of human-blood-derived monocytes to DLBCL is mediated by tumor-derived S1P.

### 3.3. S1P Promotes the Migration of M1 and M2 Macrophages to DLBCL

**Figure 3:**
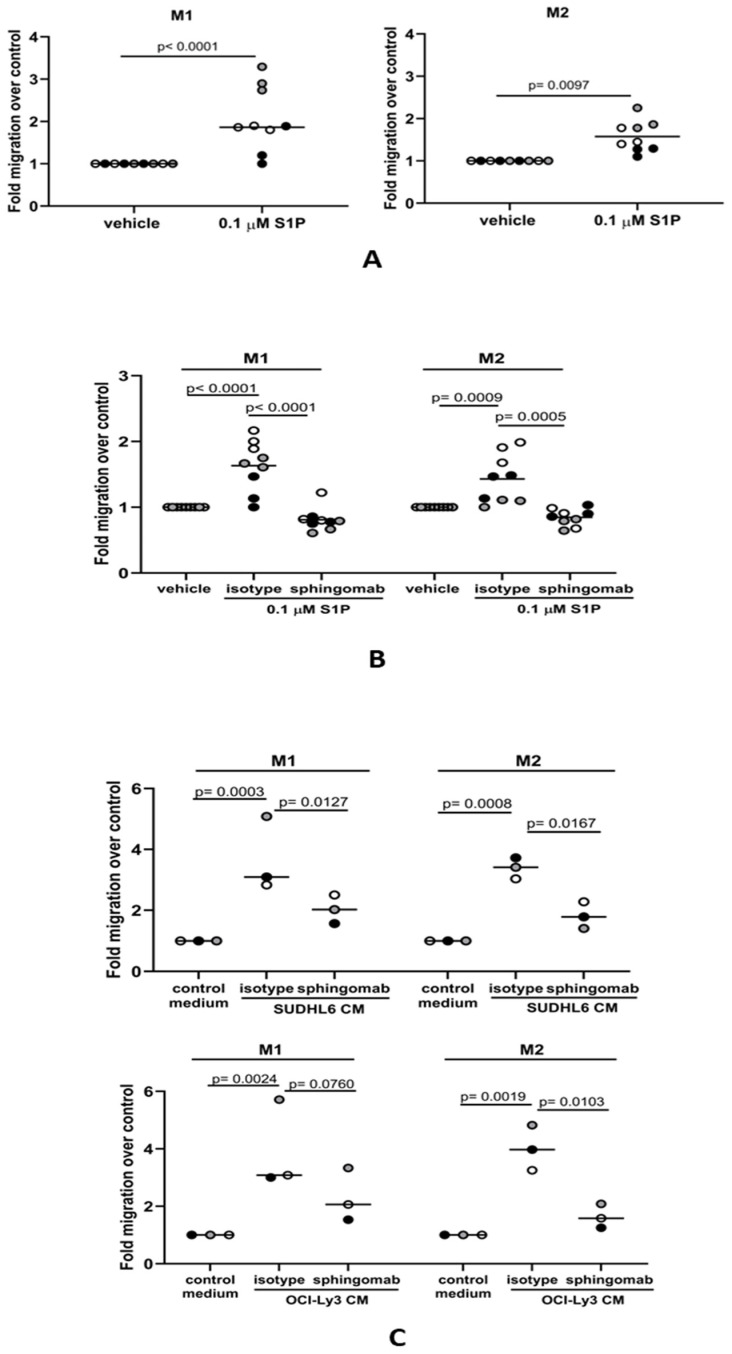
S1P mediates the migration of macrophages to DLBCL-conditioned media in vitro. (**A**) Transwell assay showing that the migration of M1 and M2 macrophages is significantly increased in the presence of 0.1 µM S1P. (**B**) S1P-induced migration of both M1 and M2 macrophages was blocked by sphingomab (0.1 µg/mL). (**C**) The migration of M1 and M2 macrophages to conditioned media (CM) from DLBCL cell lines was inhibited by sphingomab. Data show three (**A**,**B**) and one (**C**) technical replicate(s) for three individual donors and are representative of three independent experiments. The different donors are indicated by grey, black, and white circles; data means are shown by solid bars. *p*-values were calculated using a paired *t*-test (**A**) and two-way ANOVA (**B**,**C**).

Mature macrophages present in the tissues surrounding DLBCL might also be recruited to the tumor site. Therefore, we asked if macrophages could also migrate to media conditioned by DLBCL tumor cells and if this was S1P-dependent. Conventionally, tissue-resident macrophages represent a spectrum of differentiated phenotypes that can be broadly classified into two major groups: pro-inflammatory M1 macrophages important in host defenses against pathogens, including phagocytosis and the secretion of microbicidal molecules, and M2 macrophages, which are involved in the resolution phase of inflammation and tissue repair. We first polarized CD14+ blood-derived monocytes to either M1- or M2-like phenotypes using GM-CSF and M-CSF, respectively. In keeping with previous reports, GM-CSF-polarized M1-like macrophages were CD68+CD163− and moderately CD206+, whereas M-CSF-polarized M2-like macrophages were CD68+CD163+CD206+ (Figure S1A–C). To confirm the phenotype of polarized cells, we treated them with LPS for 24 h and measured cytokine levels in the conditioned media. As expected, GM-CSF-polarized macrophages had a characteristic M1 cytokine profile with low IL-10 and high IL-12 (p70) secretion, and M-CSF-polarized macrophages had a typical M2 macrophage cytokine profile with high IL-10 and low IL-12 (p70) (Figure S1D). Next, we studied the migration of polarized macrophages to S1P. We found that migration of both M1 and M2 macrophages was significantly increased in the presence of S1P (Figure 3A), an effect that was also efficiently blocked by sphingomab (Figure 3B). We next examined if the S1P generated by DLBCL cell lines could also promote the migration of macrophages. To do this, we studied the migration of macrophages to conditioned media from either SUDHL6 (GCB) or OCI-Ly3 (ABC) DLBCL cell lines in the presence or absence of sphingomab. Figure 3C shows that migration of both M1 and M2 macrophages was increased by exposure to conditioned media from both cell lines and that this was blocked by sphingomab. We conclude that macrophage migration to DLBCL cells is S1P-dependent.

### 3.4. The S1P-Mediated In Vitro Migration of Monocytes and Macrophages Is S1PR1-Dependent

**Figure 4:**
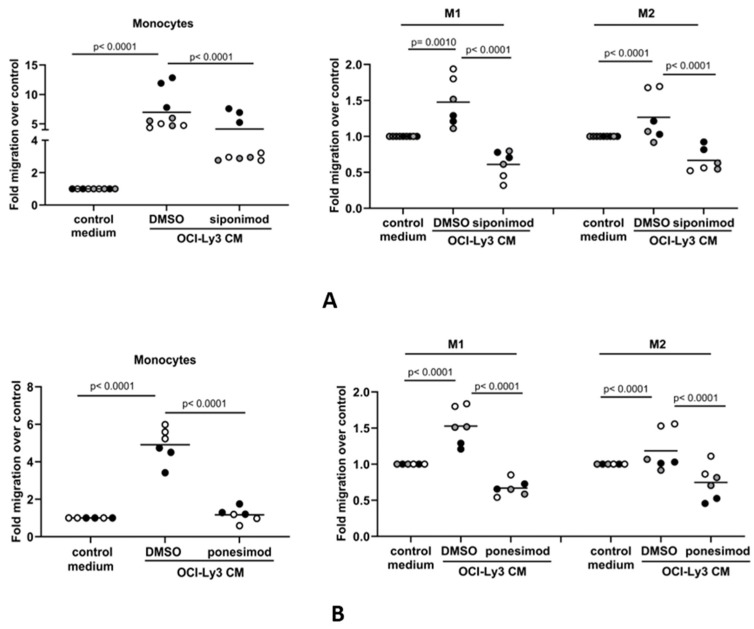
The S1P-mediated in vitro migration of monocytes and macrophages is S1PR1-dependent. Conditioned media (CM) from OCI-Ly3 cells was used as a chemoattractant in transwell migration assays with monocytes or M1 and M2 macrophages. (**A**) Monocyte migration to OCI-Ly3 CM was partially blocked by pre-treatment with 100 nM of the S1PR1 inhibitor siponimod. The increased migration of M1 and M2 macrophages to CM from OCI-Ly3 cells was also blocked by siponimod. (**B**) The migration of monocytes and macrophages to OCI-Ly3 CM was also inhibited in the presence of 100 nM of the S1PR1-specific inhibitor ponesimod. Data show two or three donors (indicated by different colors as grey, black, and white circles) in two or three technical replicates (indicated by the same color) and are representative of three independent experiments. Data means are shown by solid bars. *p*-values were calculated using two-way ANOVA.

We next explored if S1P-mediated migration of monocytes and macrophages to DLBCL conditioned media occurs via the S1PR1 receptor, which is the major S1P receptor responsible for promoting immune cell migration [58,59]. Flow cytometry confirmed that monocytes and both macrophage subsets expressed surface S1PR1 protein, although levels were decreased in both M1 and M2 macrophages compared with monocytes (Figure S2A). It was notable that, by qRT-PCR, the mRNA levels of S1PR3 and S1PR5 were also found to be significantly decreased in differentiated macrophages, while that of S1PR2 was significantly increased (Figure S2B). We pre-treated monocytes and macrophages with 100 nM of the S1PR1 inhibitor siponimod or a vehicle before measuring their migration to OCI-Ly3-conditioned media. Siponimod significantly reduced monocyte and macrophage migration to conditioned media (Figure 4A). Because siponimod inhibits both S1PR1 and S1PR5 [60,61], we further tested the S1PR1-specific inhibitor ponesimod [62]. Pre-treatment with 100 nM ponesimod decreased monocyte and macrophage migration to DLBCL-conditioned media (Figure 4B). Although we cannot rule out a contribution from S1PR5, we conclude that the in vitro migration of monocytes and macrophages to tumor-derived S1P is S1PR1-dependent. 

### 3.5. Siponimod Blocks the Recruitment of Macrophages to the Tumor Microenvironment in Mouse Models of DLBCL

**Figure 5:**
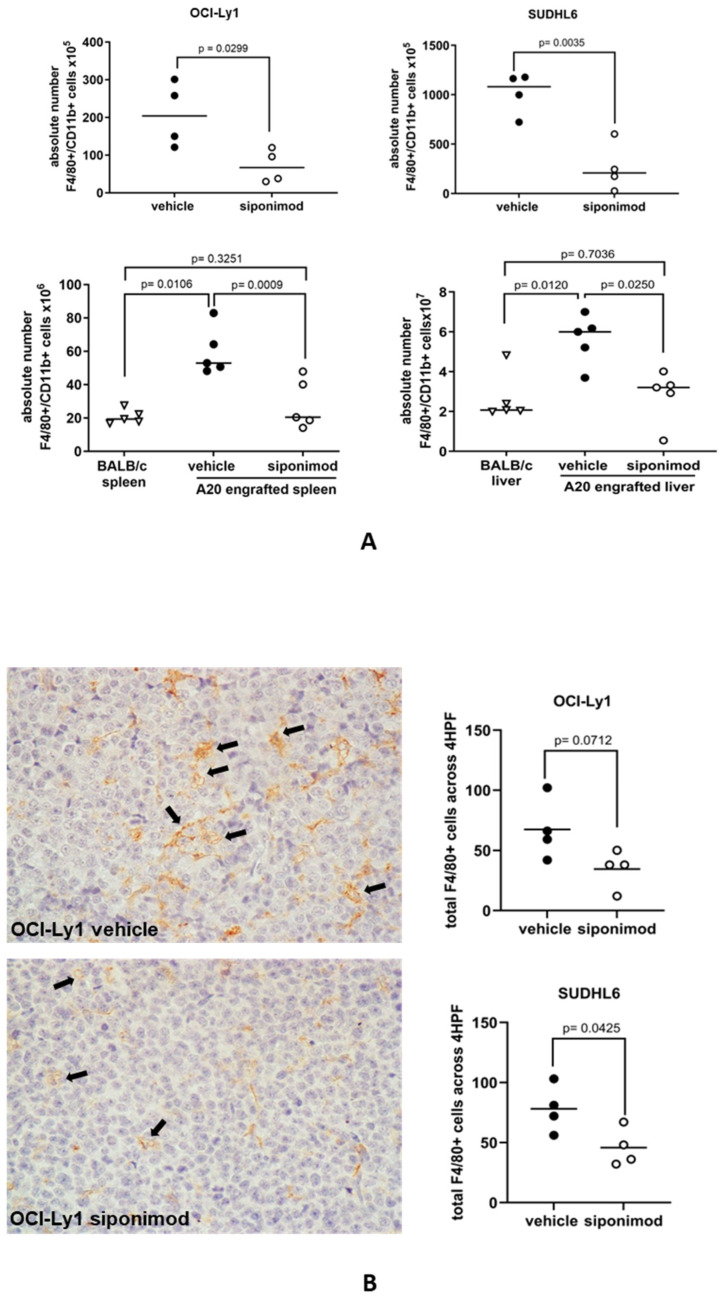
Siponimod blocks the recruitment of macrophages to DLBCL in mouse models. (**A**) Treatment with siponimod reduced the infiltration of F4/80+CD11b+ cells to OCI-Ly1 xenografts, SUDHL6 xenografts, and A20 syngeneic tumors, as measured by flow cytometry. Results shown are the mean (solid bar) of four (OCI-Ly1 and SUDHL6 xenografts) and five (A20 syngeneic tumors) mice per treatment group in each experiment. (**B**) Reduced infiltration of tumors by macrophages was confirmed by immunohistochemistry of OCI-Ly1 and SUDHL6 tumors. The left panel shows a representative example of staining in vehicle- and siponimod-treated OCI-Ly1 xenografts for the F4/80 mouse macrophage marker. Data means are shown by solid bars. *p*-values were calculated using a paired *t*-test ((**A**) top panels and (**B**)) and two-way ANOVA ((**A**) bottom panels). Absolute numbers of macrophages shown are per mg of mouse tissue.

We next studied if S1P signaling was important for the recruitment of macrophages to DLBCL in vivo. To do this, we studied the effects of siponimod on the recruitment of macrophages in mouse models of DLBCL. Mice were treated with 6 mg/kg siponimod or a vehicle as a control every other day and culled when at least one of the mice had a tumor length (longest diameter) of 1.1 cm. Flow cytometry was used to measure the absolute numbers of F4/80+CD11b+ mouse macrophages in tumor suspensions (Figure S3). We found that the number of F4/80+CD11b+ mouse macrophages was significantly reduced in both OCI-Ly1 and SUDHL6 xenografts and in the BALB/c syngeneic A20 tumors of animals treated with siponimod, compared with the tumors of animals treated with the vehicle only (Figure 5A). By immunohistochemistry, we also observed a reduction in the number of macrophages infiltrating both OCI-Ly1 and SUDHL6 tumors, but this was statistically significant only for SUDHL6 tumors, and only of borderline significance for OCI-Ly1 (Figure 5B). We conclude that siponimod can block the recruitment of macrophages to DLBCL tumors in vivo.

### 3.6. S1P Suppresses the Rituximab-Mediated Phagocytosis of DLBCL Cells by M1 Macrophages

**Figure 6:**
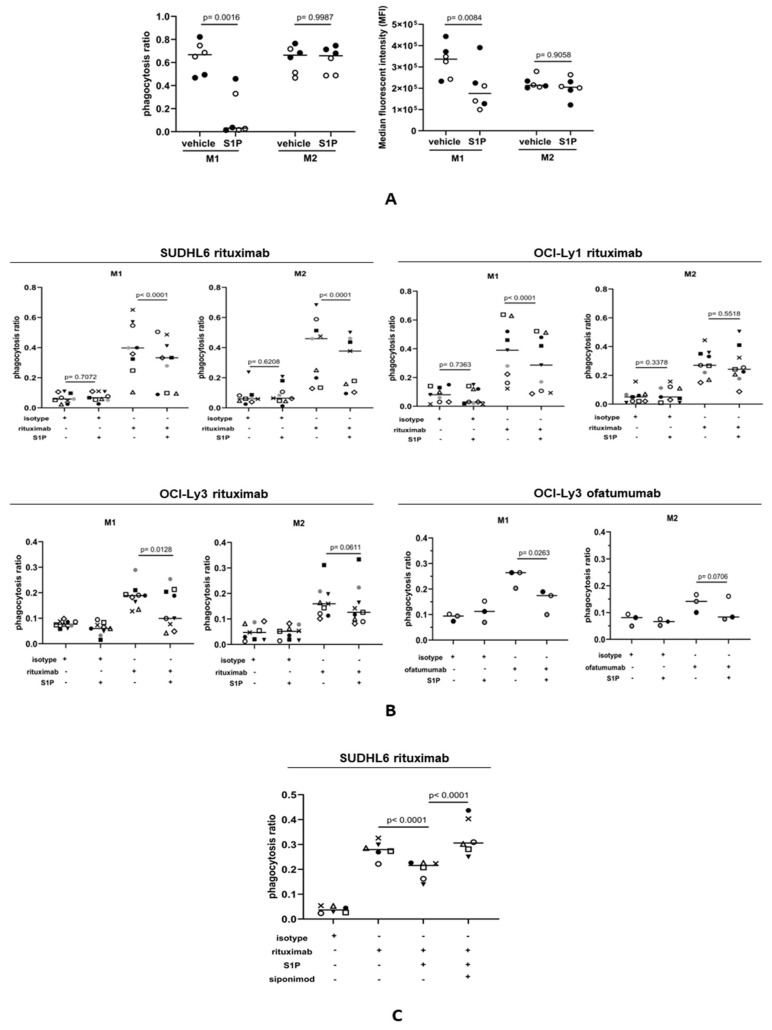
S1P suppresses the CD20-antibody-mediated phagocytosis of DLBCL cells. (**A**) Phagocytosis of FITC+ beads by M1 and M2 macrophages. The left panel shows the phagocytosis ratio calculated by dividing the number of FITC+eFluor450+ macrophages by the total number of eFluor450+ macrophages. The right panel shows the median fluorescence intensity in the same experiment. The phagocytosis of FITC+ beads by M1 macrophages, but not M2 macrophages, was significantly reduced in the presence of S1P. Data show two donors (indicated by different colors) in three technical replicates and are representative of three independent experiments. Data means are shown by solid bars. *p*-values were calculated using a paired *t*-test. (**B**) Phagocytosis ratio of M1 and M2 macrophages co-cultured with DLBCL cell lines in the presence of rituximab (1 µg/mL) ± 1 µM S1P. Flow cytometry was used to measure double-positive (CFSE+eFluor450+) macrophage populations indicating phagocytosis. There was a significant decrease in the phagocytosis of rituximab-opsonized OCI-Ly1, SUDHL6, and OCI-Ly3 cells by M1 macrophages. In contrast, only rituximab-opsonized SUDHL6 cells showed decreased phagocytosis by M2 macrophages. Three individual donors were used in three independent experiments. The results are shown for all nine donors. The anti-CD20 antibody ofatumumab (1 µg/mL) was used to confirm these effects in OCI-Ly3 cells using the same three donors as for rituximab. Data means are shown by solid bars. *p*-values were calculated using two-way ANOVA. (**C**) Phagocytosis of rituximab-opsonized SUDHL6 cells by M1 macrophages pre-treated with S1P ± siponimod. There was a decrease in phagocytosis by M1 macrophages after S1P treatment, which was reversed with siponimod. Three individual donors were used in two independent experiments. The results are shown for all six donors. Data means are shown by solid bars. *p*-values were calculated using two-way ANOVA.

Antibody-dependent cellular phagocytosis (ADCP) has been shown to be a major mediator of the therapeutic effects of rituximab [63,64]. To study the influence of S1P on ADCP, we first measured the phagocytosis of FITC+ beads by M1 and M2 macrophages stained with eFluor450 dye, adding Trypan blue to quench the signal from the FITC+ beads not engulfed by macrophages. Phagocytosis was assessed by flow cytometry. A phagocytosis ratio was calculated by dividing the number of FITC+eFluor450+ macrophages by the total number of eFluor450+ macrophages. As an additional measure of phagocytic activity, the median fluorescence intensity (MFI) was also determined for each sample. M1 and M2 macrophages were incubated with S1P or with a vehicle as a control for 1 h prior to the addition of FITC+ beads. We found that the phagocytosis of FITC+ beads by M1 macrophages, as determined by both the phagocytosis ratio and MFI, was significantly reduced in the presence of S1P (Figure 6A). In contrast, the phagocytosis of FITC+ beads by M2 macrophages was not significantly altered by S1P. We next explored if S1P also influenced the phagocytosis of rituximab-treated DLBCL cells. DLBCL lines were stained with eFluor450 cell dye and treated with rituximab or with an isotype (IgG) control. CFSE-stained macrophages were co-cultured with tumor cells and flow cytometry was used to measure double-positive (CFSE+eFluor450+) macrophage populations indicating phagocytosis. Representative plots illustrating the gating strategy are shown in Figure S4. Compared with controls, the addition of S1P significantly reduced the rituximab-mediated phagocytosis of SUDHL6, OCI-Ly1, and OCI-Ly3 cells by M1 macrophages (Figure 6B). In contrast, phagocytosis by M2 macrophages was significantly reduced only for SUDHL6 cells. To confirm this, we repeated this experiment in OCI-Ly3 cells, this time using the CD20-targeting antibody ofatumumab. We found that S1P significantly reduced the ofatumumab-mediated phagocytosis of OCI-Ly3 cells by M1, but not M2, macrophages. We conclude that S1P reduces the phagocytic capability of M1 macrophages. Finally, we explored if S1PR1 was also responsible for the S1P-mediated inhibition of phagocytosis. To do this, M1 macrophages were pre-treated with S1P ± siponimod. As before, S1P inhibited the phagocytosis of rituximab-treated SUDHL6 cells by M1 macrophages, but the addition of siponimod reversed this inhibition (Figure 6C). We conclude that inhibition of the phagocytosis of rituximab-opsonized DLBCL cells by S1P is S1PR1-dependent. 

## 4. Discussion

We have explored the effects of tumor-derived S1P on the migration and functional capacity of monocytes and monocyte-derived macrophages. Our data show that the migration of monocytes and macrophages toward media conditioned by DLBCL tumor cells can be blocked by an S1P-neutralizing antibody, showing that S1P is important for the recruitment of monocytes and monocyte-derived macrophages at tumor sites of DLBCL. In keeping with this, DLBCL cell lines have previously been shown to chemoattract blood monocytes [65]. Elevated blood monocyte counts have been suggested to be an indirect indicator of the tumor microenvironment of DLBCL, reflecting the activities of immunosuppressive peripheral blood monocytes recruited by lymphoma cells that undergo differentiation into macrophages with subsequent activity in the tumor [66]. Our observations are also supported by the meta-analysis, which revealed a striking correlation between the expression of *SPHK1* (which we used as a surrogate of S1P levels) and the expression of macrophage-associated genes, including *CD68*. This correlation was not explained by the expression of SPHK1 by macrophages since as we have described previously, SPHK1 is mostly expressed at the protein level in the tumor cells of DLBCL [44]. 

We showed that siponimod and ponesimod, inhibitors of the pro-migratory S1P receptor S1PR1, reduced the migration of monocytes and macrophages to DLBCL-conditioned media. Siponimod also reduced the migration of mouse macrophages to DLBCL tumors in animal models, including xenografts of two DLBCL lines and the A20 syngeneic DLBCL line. The observation that the migration of monocytes and macrophages both in vitro and in animal models of DLBCL is dependent upon S1PR1 is in accordance with previous data showing that S1PR1 is pro-migratory for peritoneal macrophages and for bone-marrow-derived macrophages stimulated by S1P-enriched extracellular vesicles during hepatic injury [67,68]. S1PR1 has also been shown to be essential for post-inflammatory macrophage emigration as shown in a mouse model in which the macrophage-specific deletion of S1PR1 reduced emigration of macrophages from the site of inflammation [69].

The primary mode of killing of B cell lymphoma cells by unconjugated therapeutic CD20 monoclonal antibodies, including rituximab and ofatumumab, appears to be through ADCP. Thus, CD20-antibody-mediated clearance of circulating B cells in mice is dependent on ADCP by macrophages, with a minimal contribution of NK-cell-mediated cytotoxicity [19,70,71,72,73,74]. The primary role of ADCP in the clearance of opsonized target cells is further supported by data showing that human-monocyte-derived macrophages can execute the rapid phagocytosis of multiple antibody-ligated chronic lymphocytic leukemia (CLL) cells [75]. Given the importance of ADCP in mediating CD20-antibody-mediated killing, we examined the effect of S1P on the capacity of macrophages to phagocytose DLBCL cells in vitro. We showed that S1P reduced the phagocytosis of DLBCL cells by macrophages, an effect that was mainly restricted to M1-like cells and observed with both rituximab and ofatumumab. Recent studies [76], including our own unpublished report (Fennell et al., manuscript in preparation), show substantial variability in the relative frequencies of M1 and M2 macrophages between different DLBCL tumors. This variability may be responsible for some of the differential sensitivity to rituximab observed in patients. We further showed that this inhibitory effect could be reversed with siponimod, indicating that reduced phagocytic activity is mediated primarily by S1PR1. 

Previous studies suggest that CD20-antibody-induced ADCP is a finite-capacity cytotoxic mechanism and that increasing mAb concentrations above the level required to achieve maximum efficacy will not improve the clinical outcome for patients [75]. Our data demonstrate an important S1P–S1PR1-mediated interaction between DLBCL cells and macrophages in the tumor microenvironment that impacts the efficacy of CD20 antibody immunotherapy. Alternative approaches that relieve the inhibitory mechanisms, in this case S1PR1 signaling, to allow for more efficient ADCP, could improve outcomes for patients with primary refractory disease and for those who relapse after chemo-immunotherapy.

S1PR1 inhibitors, such as siponimod and ponesimod, are already in clinical use for the treatment of multiple sclerosis, and there is potential for their application in modulating responses to CD20 antibody immunotherapy in lymphoma patients [77,78,79]. It has already been suggested that S1PR1 inhibitors could be used to treat patients with DLBCL. Thus, in an earlier study it was reported that FTY720, an antagonist of S1PR signaling that is already approved for the treatment of patients with multiple sclerosis, can inhibit S1PR1 expression, downregulate STAT3 activity, and inhibit the growth of DLBCL cells both in vitro and in vivo [41,80]. More recently, we have shown that the S1PR1 inhibitor siponimod, when used as a single agent, can reduce angiogenesis and tumor growth in an animal model of angiogenic DLBCL [44]. Our new data suggest that future studies should focus on investigating the potential therapeutic effects of combining S1P signaling antagonists following chemotherapy-induced recruitment of macrophages and before CD20 antibody therapy. 

## 5. Conclusions

In summary, we show that DLBCL-derived S1P has opposing effects on the migration and phagocytic functional capacity of monocytes and macrophages. Not only are these effects likely to be crucial for our understanding of DLBCL biology but they could also have an impact on therapeutic responses to chemo-immunotherapy. 

## Data Availability

Data are contained within the article and Supplementary Materials.

## Supplementary Materials

The following supporting information can be downloaded at: https://www.mdpi.com/article/10.3390/cancers16030574/s1, Figure S1: In vitro polarization of CD14+ monocytes to M1 and M2 macrophages; Figure S2: S1PR1 expression by flow cytometry; Figure S3: Gating strategy for analysis of mouse macrophages in tumors by flow cytometry; Figure S4: Phagocytosis assay: experimental design and flow cytometry gating strategy; Supplementary Materials and Methods: qRT-PCR.
