# Supplementary material for: The Oncogenic Lipid Sphingosine-1-Phosphate Impedes the Phagocytosis of Tumor Cells by M1 Macrophages in Diffuse Large B Cell Lymphoma
Source: Cancers (Basel). 2024 Jan 29;16(3):574. doi: 10.3390/cancers16030574 (PMC10854869; doi:10.3390/cancers16030574)
Supplement: Supplementary file 1 [file cancers-16-00574-s001.zip › Supplementary figures PERRY 28 January 2024.pdf]

Figure S1: In vitro polarization of CD14+ monocytes to M1 and M2 macrophages

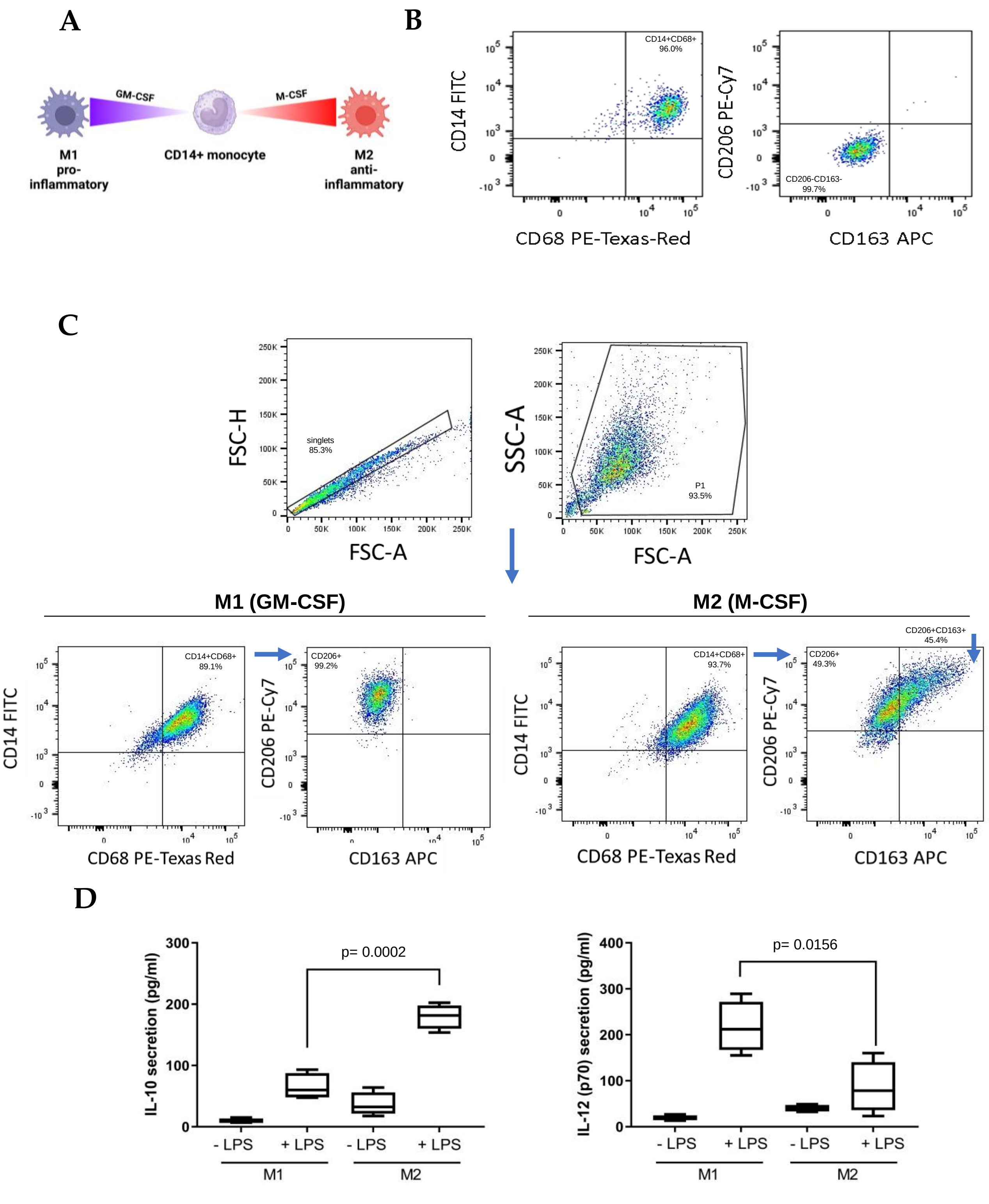

Figure S1: In vitro polarization of CD14+ monocytes to M1 and M2 macrophages

(A) GM-CSF and M-CSF were used to polarize monocytes. **(B)** Representative dot plots of CD14+ isolated cells on day 0 show high CD68 and low CD163 and CD206 expression. **(C)** Representative dot plots of M1 and M2 polarized macrophages for CD163 and CD206 expression. **(D)** ELISA results from four independent donors for IL-10 and IL-12p70 showing that M2 macrophages had higher levels of IL-10 and M1 macrophages higher levels of IL-12p70. Cytokines were measured from the culture media of M1 and M2 polarized macrophages treated for 24 h +/- LPS.

Figure S2: S1P receptor expression in monocytes, M1 and M2 macrophages

A

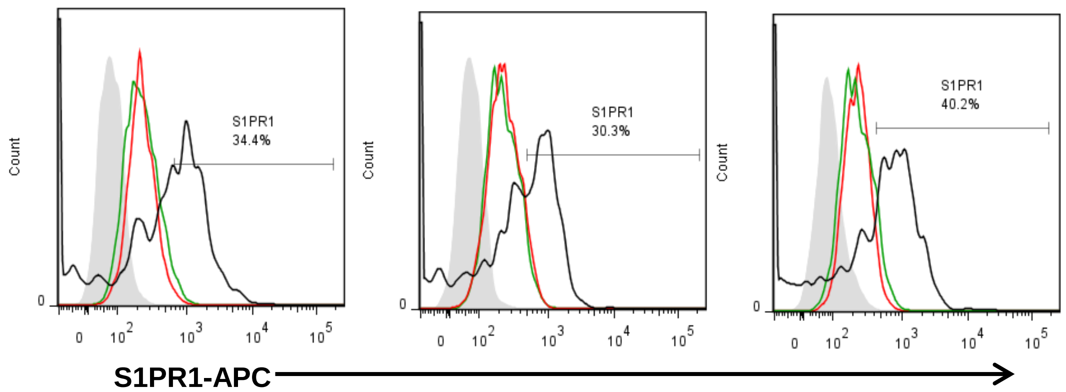

Populations	Donor 1	Donor 2	Donor 3
M1	17.2%	16.9%	18.9%
M2	18.5%	16.7%	18.7%
monocytes	34.4%	30.3%	40.2%

B

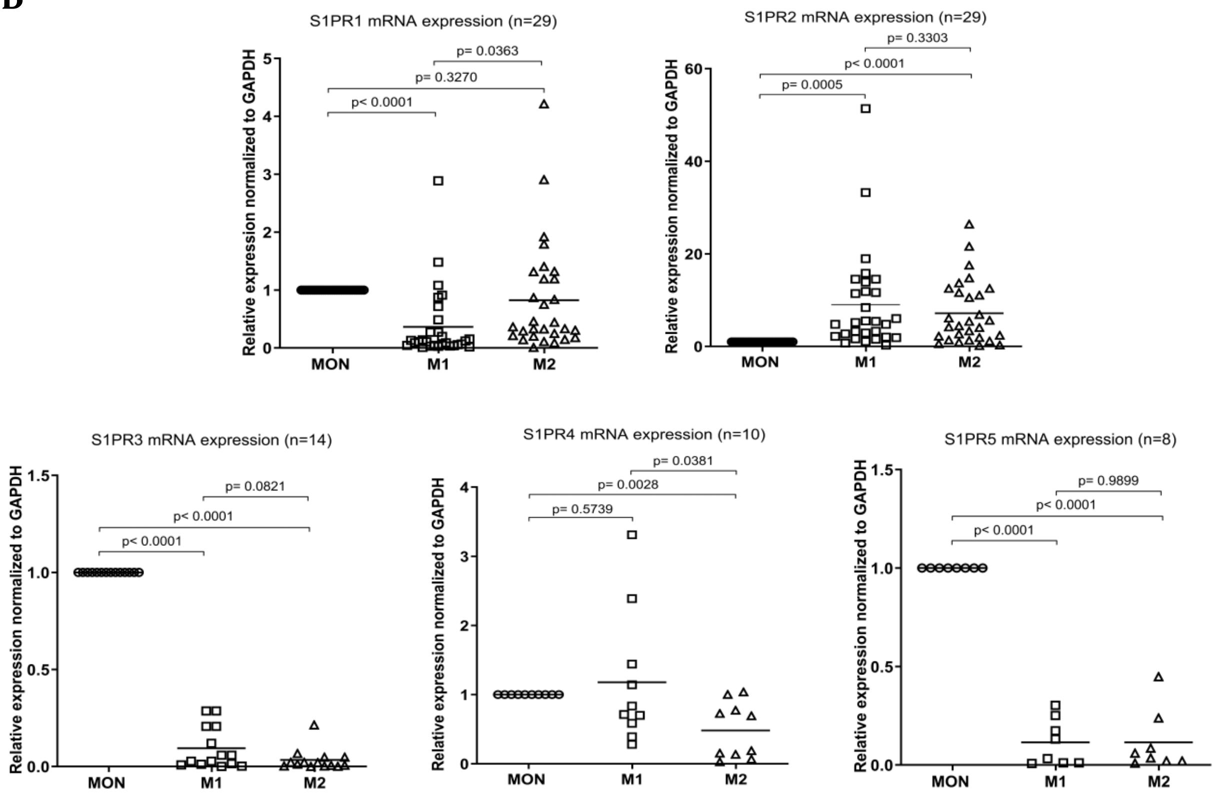

Figure S2: S1P receptors expression in monocytes, M1 and M2 macrophages
(A) S1PR1 protein expression by flow cytometry. All monocytes, M1 and M2 macrophages expressed surface S1PR1 protein. **(B)** qRT-PCR for S1P receptors (S1PR1-5) mRNA levels in monocytes (MON), M1 and M2 macrophages. Details of qRT-PCR are shown in Supplementary Materials and Methods.

Figure S3: Gating strategy for analysis of mouse macrophages in tumors by flow cytometry

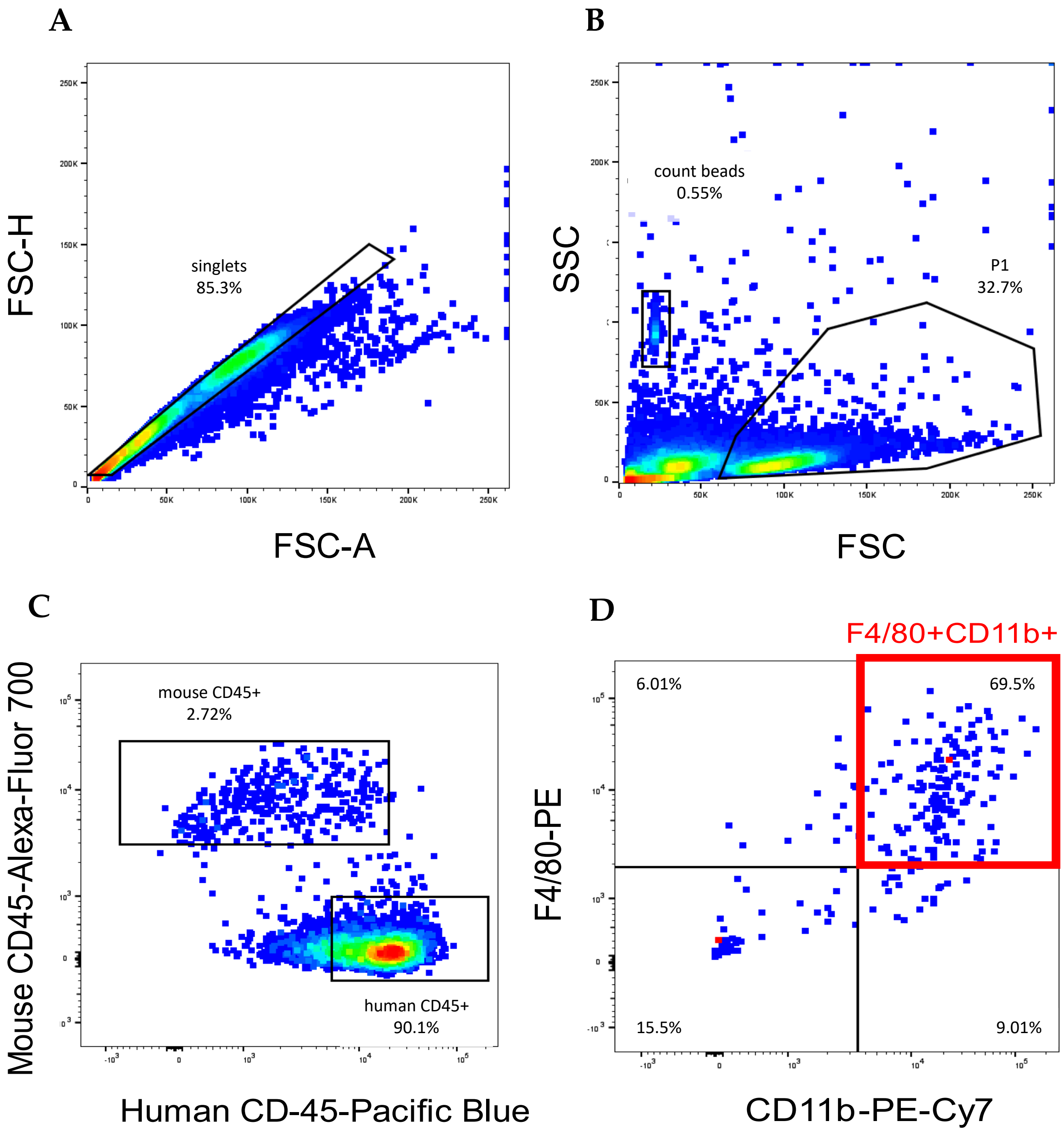

Figure S3: Gating strategy for analysis of mouse macrophages in tumors by flow cytometry
(A) Singlets were gated **(B)** then Count Bright beads and live cells were gated on FSC/SSC. **(C)** These cells were then analyzed for mouse CD45-AlexaFluor 700 vs human CD45-Pacific blue. **(D)** The mouse CD45+/human CD45- cells were gated for F4/80-PE and CD11b-PECy7.

Figure S4: Phagocytosis assay, experimental design and flow cytometry gating strategy

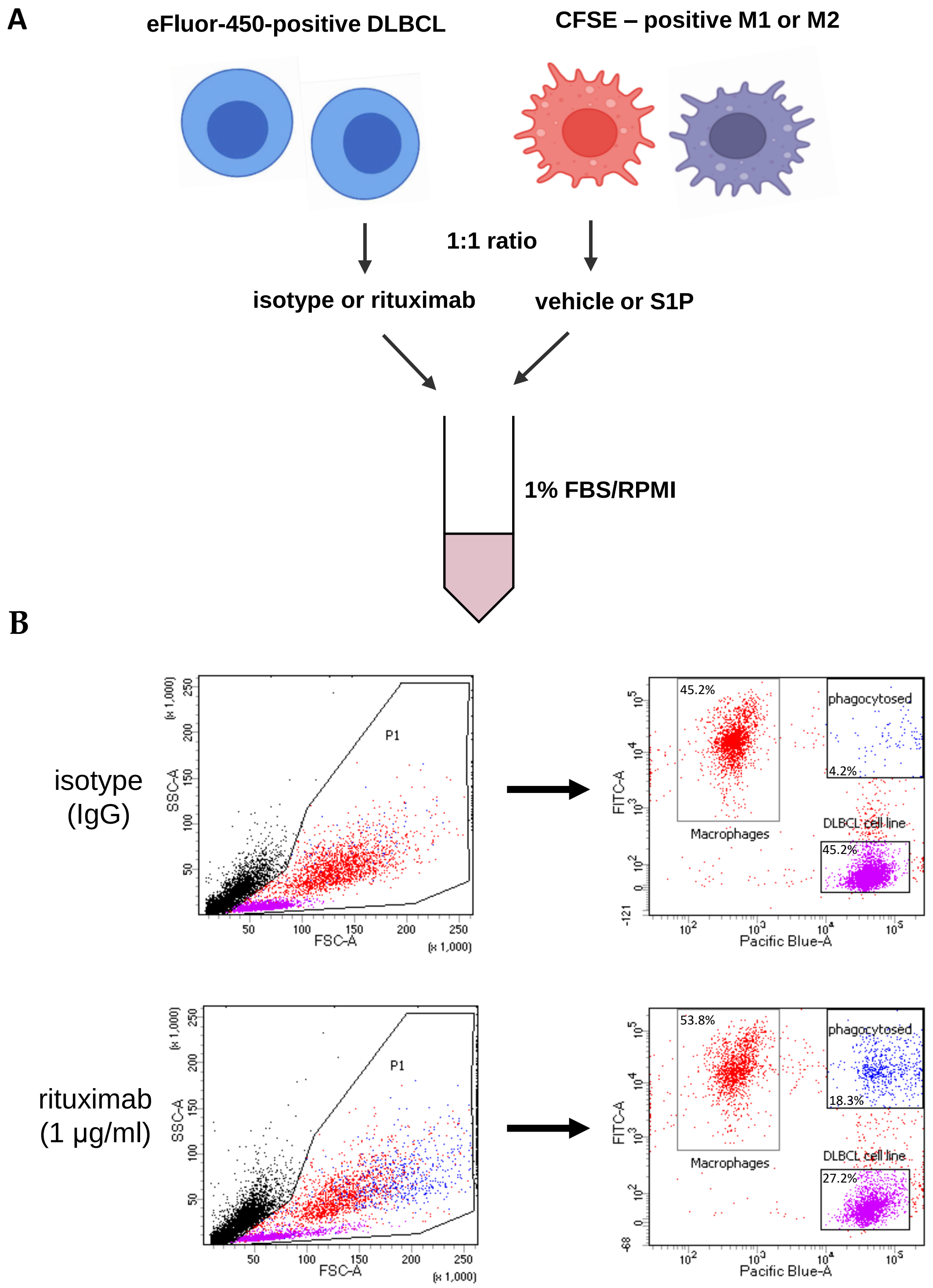

Figure S4: Phagocytosis assay, experimental design and flow cytometry gating strategy
(A) Macrophages (CFSE-positive) were pre-treated with 1 µM S1P or vehicle for 1 h and DLBCL cell lines (eFluor450-positive) were pre-treated with 1 µg/ml rituximab or isotype control for 2 h and then co-cultured for 1.5 h. **(B)** Samples were visualized on BD LSRII and analyzed with FlowJo v10. Isotype control treated DLBCL cells had a lower level of phagocytosis compared to rituximab treated DLBCL cells.
