# Supplementary material for: The Oncogenic Lipid Sphingosine-1-Phosphate Impedes the Phagocytosis of Tumor Cells by M1 Macrophages in Diffuse Large B Cell Lymphoma
Source: Cancers (Basel). 2024 Jan 29;16(3):574. doi: 10.3390/cancers16030574 (PMC10854869; doi:10.3390/cancers16030574)
Supplement: Supplementary file 1 [file cancers-16-00574-s001.zip › Supplementary methods PERRY 28 January 2024.pdf]

RNA isolation, cDNA synthesis and quantitative real time polymerase chain reaction (qPCR)

RNA was isolated with RNeasy Mini kit including genomic DNA removal using RNase-Free DNase Set (Qiagen Ltd, Manchester, UK) according to the manufacturer's protocol. The RNA concentration was quantified on a NanoDrop ND-1000 spectrophotometer (Thermo Fisher Scientific Inc, Waltham, MA, USA) and stored at -80°C.

Complementary DNA (cDNA) was synthesised with qScript™ cDNA SuperMix (QuantaBio, Beverly, MA, USA) using a Veriti Thermal Cycler (Applied Biosystems; Life Technologies Ltd, Paisley, UK) under following conditions: 5 min at 25°C, 30 min at 42°C and 5 min at 85°C and stored at -20°C.

Gene transcripts were quantified by qPCR a with commercial gene expression assays using the ABI Prism 7700 sequence detection system (Applied Biosystems, Thermo Fisher Scientific). Each reaction consisted of 5 µl of diluted cDNA (1:10 with nuclease-free water), 10 µl of FastStart Universal Probe Master Mix (Roche Diagnostics, Basel, Switzerland), 1 µl of 20x primer/probe of target gene, 1 µl of 20x primer/probe endogenous control (Taqman, Applied Biosystems) and 3 µl of nuclease free water. All reactions were run in triplicate including a water only (negative control) for each primer-probe gene of interest. The amplification was performed under the following thermal-cycling conditions: enzyme activation at 50°C for 2 min, denaturation at 95°C for 10 min followed by 40 cycles of amplification, at 95°C for 15 s and extension at 60°C for 1 min. The relative gene expression was calculated by delta (ΔΔ) Ct method and target gene values were normalized against an endogenous control (glyceraldehyde 3-phosphate dehydrogenase, GAPDH). The normalized values are shown relative to the reference sample that was set to a relative quantity value of 1. All reactions were run in triplicate and results are shown as the mean of the three replicates. Details of Taqman primer/probe qPCR assays are shown in the table below.

List of Taqman primer/probe qPCR assays

**Gene symbol**	**Assay ID**
**S1PR1**	Hs01922614_s1
**S1PR2**	Custom assay<br>5′ primer: GTGCTAGGCGTCTTTATCGTC<br>3′ primer: GTAGTGGGCTTTGTAGAGGATC<br>Probe: AGGAGTGGACGGGACAGGCATA
**S1PR3**	Hs00245464_s1
**S1PR4**	Hs02330084_s1
**S1PR5**	Hs00928195_s1
**GAPDH**	Hs02758991
